# Dosimetric evaluation of incorporating the revised V4.0 calibration protocol for breast intraoperative radiotherapy with the INTRABEAM system

**DOI:** 10.1002/acm2.12807

**Published:** 2020-02-10

**Authors:** Mubin Y. Shaikh, Jay Burmeister, Robin Scott, Lalith K. Kumaraswamy, Adrian Nalichowski, Michael C. Joiner

**Affiliations:** ^1^ Department of Radiation Oncology Rochester Regional Rochester NY USA; ^2^ Wayne State University School of Medicine Gershenson Radiation Oncology Center Barbara Ann Karmanos Cancer Institute Detroit MI USA; ^3^ Hunstville AL USA; ^4^ Department of Radiation Medicine Roswell Park Cancer Institute Buffalo NY USA; ^5^ Department of Oncology Gershenson Radiation Oncology Center Wayne State University Detroit USA

**Keywords:** INTRABEAM, breast intraoperative radiotherapy, linear‐quadratic model, TARGIT, V4.0, spherical applicators

## Abstract

In breast‐targeted intraoperative radiotherapy (TARGIT) clinical trials (TARGIT‐B, TARGIT‐E, TARGIT‐US), a single fraction of radiation is delivered to the tumor bed during surgery with 1.5‐ to 5.0‐cm diameter spherical applicators and an INTRABEAM x‐ray source (XRS). This factory‐calibrated XRS is characterized by two depth‐dose curves (DDCs) named "TARGIT" and "V4.0.” Presently, the TARGIT DDC is used to treat patients enrolled in clinical trials; however, the V4.0 DDC is shown to better represent the delivered dose. Therefore, we reevaluate the delivered prescriptions under the TARGIT protocols using the V4.0 DDC. A 20‐Gy dose was prescribed to the surface of the spherical applicator, and the TARGIT DDC was used to calculate the treatment time. For a constant treatment time, the V4.0 DDC was used to recalculate the dosimetry to evaluate differences in dose rate, dose, and equivalent dose in 2‐Gy fractions (EQD2) for an α/β = 3.5 Gy (endpoint of locoregional relapse). At the surface of the tumor bed (i.e., spherical applicator surface), the calculations using the V4.0 DDC predicted increased values for dose rate (43–16%), dose (28.6–23.2 Gy), and EQD2 (95–31%) for the 1.5‐ to 5.0‐cm diameter spherical applicator sizes, respectively. In general, dosimetric differences are greatest for the 1.5‐cm diameter spherical applicator. The results from this study can be interpreted as a reevaluation of dosimetry or the dangers of underdosage, which can occur if the V4.0 DDC is inadvertently used for TARGIT clinical trial patients. Because the INTRABEAM system is used in TARGIT clinical trials, accurate knowledge about absorbed dose is essential for making meaningful comparisons between radiation treatment modalities, and reproducible treatment delivery is imperative. The results of this study shed light on these concerns.

## 1 INTRODUCTION

Breast intraoperative radiotherapy (BIORT) is the delivery of radiation to the tumor bed to treat neoplastic cells within the surgical margin at the time of surgery.^1^ The delivery of intraoperative radiotherapy (IORT) is made possible by mobile linear accelerators that produce electrons (3–12 MeV), high‐dose Ir‐192 after loaders, or low‐energy kilovoltage (kV) x‐ray generators such as the INTRABEAM^®^ (Carl Zeiss Surgical GmbH, Oberkochen, Germany) or Xoft Axxent^®^ (Xoft Inc. is a subsidiary of iCad Inc., San Jose, CA.). This study focuses on the dosimetry of the INTRABEAM x‐ray generator, which is primarily used to deliver adjuvant BIORT for early‐stage breast cancer and is shown to be a viable alternative to whole breast irradiation (WBI) through the results of the targeted intraoperative radiation therapy A (TARGIT‐A) randomized trial.^2^, ^3^

The advantage of BIORT is reduced treatment length; enhanced patient convenience; and reduced dose to the contralateral breast, heart, and lungs.^3^, ^4^, ^5^ The efficacy of BIORT is being further investigated with ongoing national clinical trials. The TARGIT‐US registry trial (ClinicalTrials.gov Identifier: NCT01570998) is studying the efficacy and toxicity of breast INTRABEAM IORT with or without WBI. Clinical trials such as these rely on the accurate knowledge of dose distribution to perform meaningful dosimetric comparisons between radiation treatment modalities.

The INTRABEAM system is factory‐calibrated annually and characterized by two‐calibrated depth‐dose curves (DDCs) named “TARGIT” and “V4.0.” The TARGIT DDC gained its name from its use within the TARGIT‐A clinical trial.^6^ The TARGIT DDC is acquired using a Physikalisch‐Technische Werkstätten (PTW) model 23342 (volume: 0.02 cm^3^) ionization chamber (PTW, Freiburg, Germany) calibrated in exposure and introduced for treatment planning in the year 2000. The V4.0 DDC is acquired using a PTW model 34013 ionization chamber (volume: 0.005 cm^3^) calibrated in terms of air‐kerma and was introduced in 2016 in conjunction with the Zeiss water phantom.^7^ The PTW 23342 and PTW 34013 chambers differ with exposure and air‐kerma calibration schemes, respectively. Because of their cavity volume differences, these two chambers require distinct chamber holders and have different effective points of measurement. Consequently, at depths proximal to the x‐ray source (XRS), the TARGIT and V4.0 DDCs report differences in dose rate.^8^ Because greater differences in dose rate are shown at depths proximal to the XRS and a smaller spherical applicator surface is more proximal to the XRS, there is a greater concern of dose accuracy with the use of smaller spherical applicator sizes. The TARGIT DDC is maintained to ensure prescription consistency between TARGIT‐A and current clinical trials (i.e., TARGIT‐B, TARGIT‐E, TARGIT‐US), while the V4.0 DDC is used to perform calibration consistency checks with the Zeiss water phantom.

When compared with film and ion chamber measurements, the TARGIT DDC underestimates the delivered dose by 14–80%, while the V4.0 DDC difference ranges 1–5%.^7^ Even though the TARGIT DDC underestimates the delivered dose, it is used in treatment delivery for patients enrolled in TARGIT clinical trials. The purpose of this study is to reevaluate the dose, dose rate, and equivalent dose in 2‐Gy fractions (EQD2) for patients enrolled in TARGIT clinical trials using the V4.0 DDC.

## 2 MATERIALS AND METHODS

### 2.1 INTRABEAM system

The INTRABEAM system consists of a miniature XRS attached to a counterbalanced‐mobile‐floor stand with six degrees of freedom. This stand helps position the XRS within the patient for radiation treatment delivery. When a 50‐kV accelerating voltage is applied across the x‐ray tube, a beam of electrons is accelerated through a drift tube (10.0‐cm length and 0.32‐cm diameter) toward a thin gold target to produce x‐rays. The vendor of the INTRABEAM system reports its x‐ray beam to have these qualities: 50‐kV, 20.4‐keV effective energy (E_eff_), and 0.64 mm of aluminum (Al) half‐value layer (HVL) at 1‐cm depth in water.^9^ To treat the lumpectomy cavity, a rigid‐water‐equivalent plastic spherical applicator, within the range of 1.5‐ to 5.0‐cm diameter, is attached and centered to the x‐ray probe and then inserted into the lumpectomy cavity.^10^ For the ≤ 3.0‐cm diameter spherical applicators, an Al filter is incorporated into the spherical applicator design to remove low‐energy photons from the treatment spectrum.^11^ This Al filter increased treatment delivery time because it reduced the dose rate at the surface of the spherical applicator.^10^ The bodies of the > 3‐cm diameter spherical applicators remove low‐energy photons from the treatment spectrum; thus, they do not require a filter.^12^

### 2.2 Calibration consistency check

We performed a calibration consistency check of the manufacturer‐provided calibrated V4.0 DDC for our XRS and used a self‐shielded Zeiss water phantom. Dose rate measurements with this water phantom were directly compared with the V4.0 DDC. This phantom features a precise three‐dimensional translational stage, which offers reproducible source mounting and allows the XRS to be translated, with a precision of 0.01 cm, relative to the chamber.^7^ Within the phantom, the PTW model 34013 ionization chamber can be supported by two fixed waterproof chamber covers: one is for isotropy, and the other is for depth‐dose measurements. A DDC is generated when the XRS is translated away from the ionization chamber and a charge reading is collected with an electrometer.

The PTW model 34013 ionization chamber is calibrated with traceability to Physikalisch‐Technische Bundesanstalt (PTB) in Germany using a reference x‐ray beam with an E_eff_ = 16.4‐keV and HVL = 0.43‐mm Al, which is often referred to as “T30.” As suggested by the vendor and endorsed by other investigators, this beam quality is best matched to the INTRABEAM spectrum.^9^, ^13^ The American Association of Physicist in Medicine (AAPM) endorses PTB as an approved primary standards dosimetry laboratory and approves the use of this calibration approach for the INTRABEAM system.^14^ For depth *(z)*, the manufacturer suggests that the measurement of dose rate in water for the XRS *DR_W‐XRS_(z)* can be expressed in Equation (1).^7^

(1) $$DR_{W-XRS}\left(z\right)=Q\left(z\right)C_{\mathrm{TP}}K_{\mathrm{Elec}}K_{Q}K_{Ka\to Kw}N_{K}$$

The ionization charge *Q(z)* is collected over 60 seconds and is corrected for ambient temperature and pressure using the correction factor *C_TP_*. Additionally, the reading used these correction factors: electrometer calibration *K_Elec_*, beam quality *K_Q_*, chamber conversion *K*_Ka→Kw_, and ionization chamber calibration *N_k_*. The vendor and other investigators have endorsed the use of the T30 spectrum for the INTRABEAM spectrum; thus, the beam quality correction factor was set to unity (*K*_Q_ = 1).^7^, ^9^ The chamber conversion factor converts air‐kerma measurements to dose in water for the chamber in a T30 beam and is reported by the manufacturer on the chamber calibration certificate.^8^ For the PTW model 34013 ionization chamber, the vendor defines the effective point of measurement to be inside of the chamber's entrance foil.^15^ Thus, when the chamber is inserted into the waterproof holder, a measurement setup offset Δz using Equation (2) must be calculated.

(2) $$\Delta z=z_{H}+z_{\mathrm{GAP}}+z_{\mathrm{IC}}$$

For our phantom, the manufacturer provided the thickness of the waterproof holder, *z_H_* = 1.009 mm, and the distance between the surface of the chamber and the inside of the chamber holder wall, *z_GAP_ = *0.5 mm. The chamber certificate stated the gap between the surface of the chamber housing and the chamber reference point *z_IC_* = 0.324 mm. Thus, our Δz *=* 1.833 mm was accounted for when dose rate measurements were acquired. Measurements at 1.0‐, 2.0‐, and 3.0‐cm depths were taken three times and then averaged before they were compared with the V4.0 DDC to determine agreement accuracy. The < 1‐cm depths were not measured because they are sensitive to positioning error.^7^

### 2.3 Prescription and target dose

In the previous version of the INTRABEAM system, a single DDC (i.e., TARGIT) was installed into the control system at the time of calibration and commissioning. However, newer versions (after 2016) of the INTRABEAM system feature dual DDCs (i.e., TARGIT and V4.0). If TARGIT clinical trial patients are treated with a V4.0 DDC instead of TARGIT DDC, they would receive less dose.

To ensure prescription consistency, all TARGIT clinical trials deliver a 20‐Gy dose at the surface of the applicator using the TARGIT DDC.^3^ Equation (3) calculates the treatment time needed to deliver the prescription.

(3) $$\text{treatment time}=\frac{\text{prescription dose}}{DR_{W-XRS}\left(z\right)ATF\left(z\right)}$$

For a constant treatment time, the V4.0 DDC was used to recalculate the dosimetry and evaluate the differences in dose rate, dose, and EQD2 to the lumpectomy cavity. The dose rate at the surface of the spherical applicator is a product of the dose rate of the XRS $DR_{W-XRS}\left(z\right)$ (e.g., TARGIT or V4.0 DDC) and the applicator transfer function, *ATF(z)*. The prescription dose (i.e., 20 Gy) divided by the dose rate in Gy/minute results in the treatment time in minutes. In this study, we reported the minimum, median, and maximum dose to the ≤ 1.0‐cm depth of proximal tissue because this region of tissue most likely contained neoplastic cells.^1^, ^16^

### 2.4 Dose measurement uncertainty analysis

Our measurement setup consisted of a specialized Zeiss water phantom and a PTW model 34013 parallel plate ionization chamber. The Bureau International des Poids et Mesures (BIPM) Guide to the Expression of Uncertainty in Measurement was used to estimate the propagation of uncertainty. The cumulative measurement uncertainty $\sigma_{V4.0\left(k=1\right)}$ is expressed in Equation (4) where the coverage factor of an expected distribution is *k*, and *k = 1* is one standard deviation.

(4) $$\sigma_{V4.0\left(k=1\right)}=\sqrt{\sigma_{\mathrm{rep}}^{2}+\sigma_{\mathrm{pos}}^{2}+\sigma_{\mathrm{cal}}^{2}}$$

The standard deviation from three chamber measurements, $\sigma_{\mathrm{rep}}$, the dose uncertainty due to a chamber positioning error, $\sigma_{\mathrm{pos}}$, and the cumulative uncertainty of the chamber calibration$,\sigma_{\mathrm{cal}}$, are needed. The $\sigma_{\mathrm{pos}}$ is estimated by measuring the dose rate at ±0.01‐cm depths and taking the average of the deviation. The uncertainty stated on the chamber calibration certificate, according to guidelines of BIPM, is $\sigma_{\mathrm{cal}}$ = 2% (*k* = 1).

### 2.5 Physical dose delivery accuracy

The AAPM Task Group (TG)‐167 Report defines physical dose delivery as the agreement between calculated and delivered dose under idealized conditions (i.e., homogenous water phantom). Their report recommends a 6% tolerance and notes that calibration, dose calculation algorithms, and appropriateness of user‐selected dosimetric parameters influence delivery accuracy.^14^ For radionuclides used in brachytherapy, the United States Nuclear Regulatory Commission (USNRC) defines a reportable medical event when the total dose deviates ≥20%.^17^ The AAPM TG‐167 endorses the USNRC’s < 20% threshold and if this threshold is not met, it recommends the following options: (1) repositioning the applicator or XRSs to fulfill the written directive requirements, (2) adjusting the written directive, or (3) aborting the procedure.^14^ The AAPM TG‐167 Report extends guidelines to all innovative brachytherapy devices, including the INTRABEAM system.^14^ In this study, we will highlight situations where prescribing with the V4.0 DDC can produce a ≥ 20% dose deviation.

### 2.6 Linear‐quadratic model

To evaluate the impact that revised dosimetry will have on the biological dose, we present the percent change in EQD2, which is a function of the fractionation sensitivity parameter *α/β* and the generalized Lea‐Catcheside time factor *g*. The *g* factor is introduced to consider mono‐exponential repair kinetics during treatment delivery. In the case of a constant dose rate, the generalized Lea‐Catcheside equation can be expressed as Equation (5).

(5) $$g=\frac{2\left(\mu t-1+e^{-\mu t}\right)}{{\left(\mu t\right)}^{2}}$$

The treatment duration *t* ranges from 0.12 to 0.72 hours (approximately 7 to 43 minutes). Since the dose rate decreases with increased distance, larger applicators have increased values of *t*. The repair half‐time *T_1/2_* for the tissue is used to calculate *µ* = *ln2* ${\left(T_{1/2}\right)}^{-1}$. Note, both *t* and *T_1/2_* have units of hours. Acute or late responding tissues have a range of reported *T_1/2_* values.^18^, ^19^, ^20^ Bentzen *et al.* 19 reported *T_1/2_* values for late clinical endpoints using the data from the continuous hyperfractionated accelerated radiotherapy trial and found that the *T_1/2_* values for subcutaneous fibrosis to be 3.8 hours (95% confidence interval (CI): 2.5–4.6) and for skin telangiectasia to be 4.4 hours (95% CI: 3.8–4.9).^19^ Other investigators have reported shorter *T_1/2_* values for early responding tissues and tumors of approximately 0.5 hours.^20^ Given the wide latitude in *T_1/2_* values, an average value of the generalized Lea‐Catcheside time factor was defined with a range of 0.5‐4.4 hours.

The calculation of EQD2 using the linear‐quadratic (LQ) model^21^ is expressed in Equation (6).

(6) $$EQD2_{\alpha /\beta }=D\left(\frac{dg+\alpha /\beta }{2Gy+\alpha /\beta }\right)$$

Because IORT is delivered as a single fraction of 20‐Gy, the total dose *D* and dose per fraction *d* each equal 20‐Gy. Haviland *et al.* 22 conducted a meta‐analysis of the UK Standardisation of Breast Radiotherapy (START) trials (i.e., START‐A, START‐B, and the START pilot) by fitting the Cox proportional hazards regression models to individual patient data. One of their principal endpoints, locoregional relapse, was defined as recurrence in the breast, chest wall, ipsilateral axilla, or supraclavicular fossa. Their study reported an adjusted α/β value for locoregional relapse of 3.5‐Gy (95% CI: 1.2–5.7). While for healthy tissue endpoints, the α/β values are 3.8 Gy (95% CI: 1.8–5.7) for telangiectasia and 4 Gy (95% CI: 2.3–5.6) for breast induration.^22^ We performed calculations of EQD2 for locoregional relapse of the lumpectomy cavity and used the mean α/β value of 3.5 over 1.2–5.7 CI. Because our estimates considered a wide range of α/β values, the results are relevant to a variety of different healthy tissue endpoints. We calculated the EQD2 at the surface of the spherical applicator and at a 1.0‐cm depth and compared the biologically effective dose for the competing TARGIT and V4.0 DDCs.

## 3 RESULTS

For measurement uncertainty, Table 1 shows the standard deviation from three chamber measurements, $\sigma_{\mathrm{rep}}$ is < 0.25% and $\sigma_{V4.0\left(k=1\right)}$ is < 3.5%, for the 1.0‐, 2.0‐, and 3‐cm investigated depths. Table 2 indicates a ≤ 2.48% difference between the V4.0 calibration DDC and the PTW model 34013 ion chamber measurement. Our results are within the measurement uncertainity budget (<3.5%) shown in Table 1.

**Table 1:** Measurement uncertainty with *k = 1.*

Uncertainty type	Depth in water (cm)		
	1.0	2.0	3.0
*σ_rep (%)_*	0.21	0.18	0.12
*σ_pos (%)_*	2.84	1.38	0.75
*σ_cal (%)_*	2.00	2.00	2.00
*σ_V4.0_ (k = 1)*	3.48	2.44	2.14

**Table 2:** Comparison of V4.0 calibration depth‐dose curve (DDC) with ion chamber measurements for a single x‐ray soruce.

Depth in water (cm)	V4.0 Calibration DDC (Gy/minute)	Ion Chamber Measurement^a^ (Gy/minute)	Difference^b^ (%)
1.0	3.548	3.636	2.48%
2.0	0.510	0.520	1.96%
3.0	0.197	0.199	1.02%

a PTW model 34013 ion chamber measurement.

b The difference in % is within measurement uncertainty σ_V4.0_ (k = 1) as presented in Table 1.

**Figure 1:**
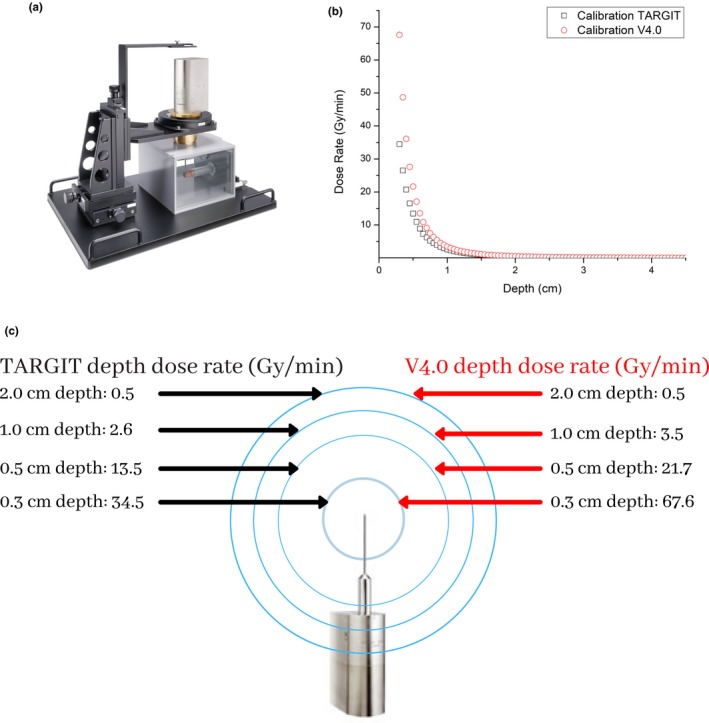
(a) The Zeiss water phantom used to acquire measurements. Copyright Carl Zeiss Meditec AG^©^ (b) A depth‐dose rate plot under TARGIT and V4.0 (c) An infographic that compares the dose rate at distances of 0.3, 0.5, 1.0, and 2.0 cm under TARGIT and V4.0 calibration.

Figure 1(a) illustrates the Zeiss water phantom used to acquire dose rate measurements. Figure 1(b) demonstrates the depth‐dose rates as a function of depth for both TARGIT calibration and V4.0 calibration. Figure 1(c) visually highlights the TARGIT and V4.0 dose rate differences for the XRS (ie, no applicator) respectively.

**Figure 2:**
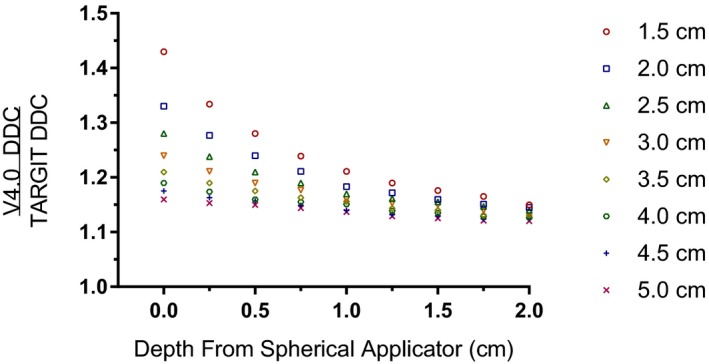
The ratio of the V4.0 DDC to the TARGIT DDC as a function of depth for all spherical applicators.

**Figure 3:**
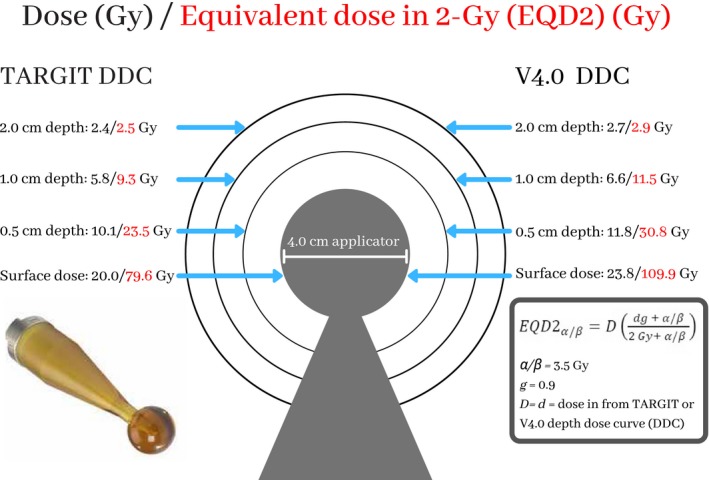
The dose distribution and EQD2 values surrounding the 4.0‐cm diameter spherical applicator

Figure 2 presents the ratio of the V4.0 DDC to the TARGIT DDC as a function of depth from the applicator surface for 1.5‐ to 5‐cm diameter spherical applicators. Figure 3 shows the difference in dose in Gy for the TARGIT and V4.0 DDCs around a 4.0‐cm diameter spherical applicator. This applicator size was chosen to offer a direct comparison to previously published dosimetric results calculated with the TARGIT DDC only.^23^ The 2.0‐, 1.0‐, and 0.5‐cm depths and surface of the 4.0‐cm diameter spherical applicator, respectively, demonstrated these doses: 2.4‐, 5.8‐, 10.1‐, and 20.0‐Gy dose and 2.5‐, 9.3‐, 23.5‐, and 79.6‐Gy EQD2 for the TARGIT DDC and 2.7‐, 6.6‐, 11.8‐, and 23.8‐Gy dose and 2.9‐, 11.5‐, 30.8‐, and 109.9‐Gy EQD2 for the V4.0 DDC.

Table 3 reports the difference in TARGIT and V4.0 dose rates at the surface of the 1.5‐ to 5.0‐cm diameter spherical applicators. The dose rate differences ranged from 43% for the 1.5‐cm diameter spherical applicator to 16% for the 5.0‐cm diameter spherical applicator. These results demonstrate that delivered dose can vary depending on the calibration method used and that all applicator sizes fail to meet the physical dose delivery accuracy of 6% recommended by the AAPM TG‐167 Report. For ≤ 3.5‐cm diameter spherical applicators, an important concern is that the < 20% dose deviation threshold for medical events was exceeded.

**Table 3:** Comparison of TARGIT and V4.0 doserate (DR) determines if dose deviation is ≥ 20%.

Spherical Applicator Diameter (cm)	Prescription Point is Applicator Surface			Dose Deviation (≥ 20%)
	TARGIT DR (Gy/minute)	V4.0 DR (Gy/minute)	Dose Rate Difference (%)	
1.5	2.755	3.934	43	Yes
2.0	1.764	2.354	33	Yes
2.5	1.228	1.568	28	Yes
3.0	0.820	1.015	24	Yes
3.5	1.080	1.307	21	Yes
4.0	0.800	0.952	19	No
4.5	0.577	0.677	17	No
5.0	0.425	0.494	16	No

Table 4 provides incomplete repair half‐time factors (i.e., *g* factor) for a range of *T_1/2_* values as a function of treatment time and applicator size. In general, when a *T_1/2_* of 4.4 hours is considered, the *g* factor approaches unity for all diameter sizes of the spherical applicators. But when the small *T_1/2_* of 0.5 hours is considered, a minimal value of 0.74 is observed for the 5.0‐cm diameter spherical applicator. To ensure a robust calculation of EQD2, we considered the average *g* factor value for each applicator as part of the EQD2 calculations.

**Table 4:** The average Lea‐Catcheside time factor is found per diameter of spherical applicator.

Spherical Applicator Diameter (cm)	20‐Gy Treatment Delivery Time (h)	Lea‐Catcheside Time Factor g Relative to Repair Half‐Time *T½* (hours)				*g Average*
		0.5	1.0	1.5	4.4	
1.5	0.12	0.95	0.97	0.98	0.99	0.97
2.0	0.19	0.92	0.96	0.97	0.99	0.96
2.5	0.23	0.90	0.95	0.97	0.99	0.95
3.0	0.35	0.86	0.92	0.95	0.98	0.93
3.5	0.27	0.89	0.94	0.96	0.99	0.94
4.0	0.38	0.85	0.92	0.94	0.98	0.92
4.5	0.52	0.80	0.89	0.93	0.97	0.90
5.0	0.72	0.74	0.85	0.90	0.96	0.86
Average	0.35	0.86	0.93	0.95	0.98	0.93

**Figure 4:**
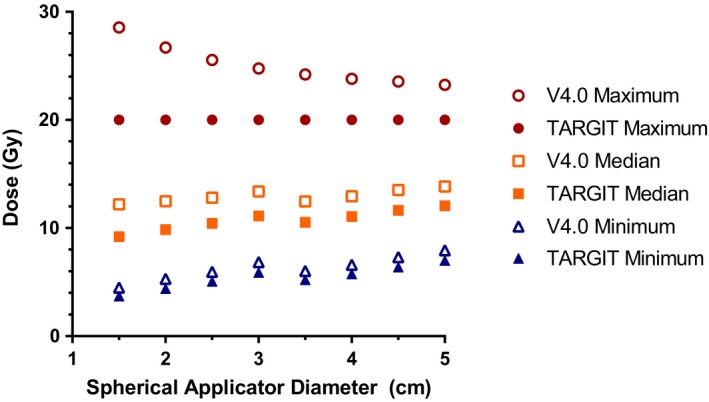
A comparison of the doses to the proximal 1‐cm depth of tissues surrounding the 1.5‐to 5.0‐cm diameter spherical applicators under TARGIT and V4.0 calibration

Figure 4 illustrates the maximum, median, and minimum dose to the proximal 1.0‐cm of the lumpectomy cavity to be consistently less with TARGIT DDC when compared to V4.0 DDC. A 20‐Gy dose is prescribed to the surface of the applicator. At the surface of the applicator, Table 5 shows a 28.6‐ to 23.2‐Gy delivered dose and a 95‐31% EQD2 difference for the 1.5‐ to 5.0‐cm diameter spherical applicators, respectively. At a 1‐cm distance from the surface of the applicator, Table 6 shows a 6.4‐ to 15.1‐Gy delivered EQD2 dose for the 1.5‐ to 5.0‐cm diameter spherical applicators, respectively. Within Table 6, the 33%, 30%, 27%, 29%, 27%, 24%, 24%, and 25% EQD2 differences were for the 1.5‐ to 5.0‐cm diameter applicators, respectively.

**Table 5:** Display of dose data at the applicator surface, when 20‐Gy is prescribed using the TARGIT DDC.

Spherical Applicator Diameter (cm)	Dose		*EQD2*					
	TARGIT^a^ (Gy)	V4.0^b^ (Gy)	*g* Factor Average	TARGIT^a^ *EQD2* c (Gy)	V4.0^b^ *EQD2* c (Gy)	*EQD2* Difference (%)	CI Range Estimate (Gy)	
							Low^d^	High^e^
1.5	20	28.6	0.97	83.3	162.5	95	124.2	258.7
2.0	20	26.7	0.96	82.5	141.4	71	108.6	223.9
2.5	20	25.5	0.95	81.8	128.5	57	99.1	202.6
3.0	20	24.8	0.93	80.4	119.8	49	92.6	188.0
3.5	20	24.2	0.94	81.1	115.5	42	89.4	181.1
4.0	20	23.8	0.92	79.6	109.9	38	85.3	171.8
4.5	20	23.5	0.90	78.2	105.3	35	81.9	164.1
5.0	20	23.2	0.86	75.3	98.9	31	77.3	153.4

a TARGIT depth‐dose curve (DDC) was used to calculate the data.

b V4.0 DDC was used to calculate the data.

c The mean α/β = 3.5 Gy was used to calculate the data for equivalent dose in 2‐Gy fractions (*EQD2*).

d The mean α/β = 5.7 Gy was used to calculate the data.

e The mean α/β = 1.2 Gy was used to calculate the data.

**Table 6:** Display of dose data at a 1‐cm depth from the applicator surface when 20‐Gy is prescribed at applicator surface.

Spherical Applicator Diameter (cm)	Dose		*EQD2*					
	TARGIT^a^ (Gy)	V4.0^b^ (Gy)	*g* Factor Average	TARGIT^a^ *EQD2* b (Gy)	V4.0^b^ *EQD2* c (Gy)	*EQD2* Difference (%)	CI Range Estimate (Gy)	
							Low^d^	High^e^
1.5	3.7	4.5	0.97	4.8	6.4	33	5.9	7.8
2.0	4.5	5.3	0.96	6.4	8.3	30	7.4	10.4
2.5	5.1	5.9	0.95	7.7	9.8	27	8.7	12.5
3.0	5.9	6.9	0.93	9.6	12.4	29	10.9	16.4
3.5	5.2	6.0	0.94	7.9	10.0	27	8.8	12.8
4.0	5.8	6.6	0.92	9.3	11.5	24	10.1	15.0
4.5	6.4	7.3	0.90	10.8	13.4	24	8.8	12.8
5.0	7.0	8.0	0.86	12.1	15.1	25	10.1	15.0

a TARGIT depth‐dose curve (DDC) was used to calculate the data.

b V4.0 DDC was used to calculate the data.

c The mean α/β = 3.5 Gy was used to calculate the data for equivalent dose in 2‐Gy fractions (*EQD2*).

d The mean α/β = 5.7 Gy was used to calculate the data.

e The mean α/β = 1.2 Gy was used to calculate the data.

In respect to the Al filter within ≤ 3.0‐cm diameters, spherical applicator variances are shown. Table 3 shows the decreased TARGIT and V4.0 dose rates when the diameter of the spherical applicator increased except for the 3.0‐ to 3.5‐cm diameter spherical applicators. Table 4 shows the increased delivery time when the diameter of the spherical applicators increased except from the 3.0‐ to 3.5‐cm diameter spherical applicators. Within Table 6, expected and delivered doses increased as the diameter of the spherical applicator increased except for the 3.0‐ to 4.0‐cm diameter spherical applicators.

## 4 DISCUSSION

The results of this study demonstrate that the dose rate, delivered dose, and EQD2 values are higher than previously expected with the maximum differences observed for the 1.5‐cm diameter spherical applicator. Because more considerable differences in dose rate are shown at depths proximal to the XRS and a smaller spherical applicator surface is more proximal to the XRS, there is an increased concern of dose accuracy with the use of smaller spherical applicator sizes. Among patients in the North American TARGIT trial, the most used spherical applicator diameters were 3.5 cm (23%) and 4 cm (35%).^24^ The 1.5‐cm diameter spherical applicator has a volume of 1.8 cm^3^ and is used for in situ ductal carcinoma cases, which tend to be less often treated in our clinic. Thus, most centers do not purchase the 1.5‐ and 2.0‐cm diameter spherical applicators.

The TARGIT‐A clinical trial assumes that the surface of the tumor bed receives a 20‐Gy dose.^3^ In contrast, our results indicate that the tumor bed could receive a 28.6‐ to 23.2‐Gy dose with the 1.5‐ to 5.0‐cm diameter spherical applicators, respectively. Thus, depending on the size of the spherical applicator, the tumor bed receives different treatment doses under TARGIT‐A and ongoing clinical trials. If the intention is to use the V4.0 calibration method and stay consistent with the TARGIT‐A trial, then an applicator‐specific prescription model needs to be introduced to the dose calculation methodology.

In Figure 3, we presented delivered dose and EQD2 doses surrounding a 4‐cm diameter spherical applicator. Previously published studies showed the dose with only the TARGIT DDC,^23^ but we presented the delivered dose and the EQD2 doses surrounding the applicator.

The current version of the INTRABEAM treatment planning software (v.4.0.1.2) permits clinicians the flexibility to prescribe 20‐Gy to the surface of the spherical applicator with either calibration DDC (e.g., TARGIT and V4.0). If the V4.0 calibration DDC was accidentally chosen for a clinical trial patient, then a dose discrepancy would be introduced. The results of this study help estimate the magnitude of this discrepancy based on spherical applicator size. Currently, the impact of dose deviations on local control for BIORT has not been presented. However, in specific clinical situations, it has been shown that dose differences of 7% can produce clinically observable outcome differences.^25^ Additionally, future studies may wish to retrospectively recalculate dose distributions using the V4.0 in order to correlate dose distribution with their outcomes.

The radiobiological response of INTRABEAM IORT has been previously explored through the use of equivalent uniform dose and relative biological effectiveness (RBE) calculations.^26^, ^27^ Using spectral Monte Carlo data for the INTRABEAM system and clinical breast tissue mixtures, White *et al*. estimated RBE values of 1.4 to 1.59 for ribs, adipose tissue, skin, and lung relative to a Cobalt‐60 reference beam. Thus, dosimetric differences have an increased biological effect. The purpose of the present work was to determine the change in EQD2 values given differences in dose rate reported by TARGIT and V4.0 DDC. Techniques such as stereotactic body radiotherapy, IORT, and high dose rate brachytherapy often deliver ≥ 10‐Gy fraction doses. In these elevated single fraction doses, the application of the simple LQ model or any of its proposed modifications is debated with conflicting views in the literature.^18^, ^28^, ^29^ However, investigators use the LQ model for IORT doses.^18^, ^30^ We found our rationale and application consistent with other investigators. The CI for α/β values derived from animal studies is narrower than for the corresponding human endpoint because of the greater number of subjects in the experimental animal studies, and the dose per fraction can be varied more systematically over a wide range of values.^18^ In our research, we have included calculations using the CI range to provide a more clinically realistic estimation of EQD2.

In the field of radiation oncology, there is precedent when dosimetry calibration standards are revised in light of new measurements. Consider the historical example of Iodine‐125 (I‐125) and Palladium‐103 (Pd‐103) radioactive sources used in the delivery of low dose rate brachytherapy. Using a standard that was set forth by the National Institute of Standards and Technology (NIST), accredited dosimetry calibration laboratories calibrated these sources. In January 2000, NIST noticed a shift in well‐chamber coefficients from select radioactive source vendors (i.e., Bebig I‐125 and IBt Pd‐103 sources) because the NIST Wide‐Angle Free‐Air Chamber was measuring the Ti K x‐ray produced in the source encapsulation. Consequently, the revised air‐kerma strength was decreased by 10.3% to address this effect.^31^ The differences in the air‐kerma strength calibration are similar to the differences in the calibration DDC presented by this study because both of these parameters directly impact patient dosimetry.

Dosimetric reviews play a pivotal role in guiding the development of future clinical trials. Consider the history of prospective clinical trials for non‐operable lung cancers pioneered by the Radiation Therapy Oncology Group (RTOG). In RTOG 0236, the decision was made to prescribe 60‐Gy in three fractions without heterogeneity corrections.^32^ Retrospective dosimetric reviews showed that the 60‐Gy prescription was equivalent to a 56‐Gy prescription with heterogeneity corrections.^32^ In response, RTOG 0613 adopted a 54‐Gy prescription and mandated heterogeneity corrections.^33^ We believe that the apparent dosimetric differences between the TARGIT DDC and the V4.0 DDC are analogous to the variations when lung treatment plans with and without heterogeneity corrections are compared. The adoption of more accurate dosimetry often takes time to be incorporated by clinical trials but nevertheless must be done to allow the most accurate evaluation of the clinical efficacy and risk from the trial.

A limitation of our study is that we have assumed that breast tissue can be estimated by a homogeneous water phantom, which is problematic because low‐energy photons are sensitive to the atomic number of the medium. White *et al.* 34 computed dose‐volume histogram metrics for an alternative 50‐kVp XRS, and they noted a decrease in dose when accounting for breast tissue inhomogeneities. In their investigation, the minimum dose to 90% of the planning target volume decreased by 4% (i.e., TG‐43 dose formalism) when compared with a similar homogenous calculation. Because heterogeneities impact both dose calculation methods similarly, this limitation does not substantially change the dosimetric differences revealed within our study.

## 5 CONCLUSION

This work demonstrated apparent dosimetric differences between the TARGIT and V4.0 calibration DDCs in the context of using spherical applicators with the INTRABEAM system for BIORT. The results from this work can be interpreted as reevaluating dose estimates using the V4.0 DDC or as highlighting the dosimetric dangers of underdosage when using the V4.0 DDC. Nevertheless, this study provides evidence that 20 Gy prescribed to a spherical applicator surface under the TARGIT and V4.0 calibration DDCs is not dosimetrically equivalent. Also, for ≤ 3‐cm diameter spherical applicators, we show ≥ 20% dosimetric differences, which does not meet the physical dose delivery accuracy threshold endorsed by the AAPM TG‐167.

## CONFLICT OF INTEREST

The authors declare no conflict of interest.
